# FingerType: One-Handed Thumb-to-Finger Text Input Using 3D Hand Tracking

**DOI:** 10.3390/s26030897

**Published:** 2026-01-29

**Authors:** Nuo Jia, Minghui Sun, Yan Li, Yang Tian, Tao Sun

**Affiliations:** 1 Key Laboratory of Symbolic Computation and Knowledge Engineering of Ministry of Education, College of Computer Science and Technology, Jilin University, Changchun 130012, China; 2 State Key Laboratory of Advanced Vehicle Integration and Control, China FAW Group Co., Ltd., Changchun 130013, China; 3 School of Computer, Electronics and Information, Guangxi University, Nanning 530004, China; 4 Changchun Institute of Optics, Fine Mechanics and Physics, Chinese Academy of Sciences, Changchun 130033, China

**Keywords:** virtual reality, text entry, bare-hand input, one-handed interaction, thumb-to-finger gestures, mid-air input

## Abstract

We present FingerType, a one-handed text input method based on thumb-to-finger gestures. FingerType detects tap events from 3D hand data using a Temporal Convolutional Network (TCN) and decodes the tap sequence into words with an n-gram language model. To inform the design, we examined thumb-to-finger interactions and collected comfort ratings of finger regions. We used these results to design an improved T9-style key layout. Our system runs at 72 frames per second and reaches 94.97% accuracy for tap detection. We conducted a six-block user study with 24 participants and compared FingerType with controller input and touch input. Entry speed increased from 5.88 WPM in the first practice block to 10.63 WPM in the final block. FingerType also supported more eyes-free typing: attention on the display panel within ±15° of head-gaze was 84.41%, higher than touch input (69.47%). Finally, we report error patterns and WPM learning curves, and a model-based analysis suggests improving gesture recognition accuracy could further increase speed and narrow the gap to traditional VR input methods.

## 1. Introduction

As Virtual Reality (VR) hardware and software mature, VR is being used in a wider range of real-world and everyday scenarios. Two-handed interaction is intuitive but assumes both hands are available. Real-world VR usage often contradicts this. Users frequently hold objects like tools, drinks, or bags, or must hold a handrail for stability in mobile environments. In these asymmetric scenarios, forcing two-handed input disrupts the user’s primary task. Therefore, effective one-handed text entry is not merely an alternative but a necessity for always-available computing. External devices such as keyboards, touchscreens, and wearable sensors [1,2] can provide VR input, but they may reduce portability and comfort. They also add practical overhead (e.g., donning/doffing), and users may simply forget to bring them, which limits everyday usability [3]. Alternatives such as speech recognition (e.g., Google Glass, Microsoft HoloLens), in-air typing [4], and other non-traditional methods (e.g., eye tracking [5] and swipe gestures [6]) can reduce reliance on physical hardware, but often raise social discomfort, require sustained arm motion that increases fatigue [7], and increase learning costs due to unfamiliar interaction paradigms. Therefore, there is a need for a convenient and adaptable VR text-entry method that supports one-handed, bare-hand use with low visual demand.

Hand tracking and gesture-based input show promise in addressing these issues. In particular, thumb-to-finger interactions are intuitive and benefit from intrinsic tactile feedback. Huang et al. [8] showed that thumb-to-finger contact enhances physical control, and haptic feedback helps users better perceive the thumb’s position, improving accuracy. Pseudo- and self-haptic cues further support learning and interaction [9]. Building on this, researchers explored hand input using data gloves [1,10] and finger-based gestures [11,12,13]. Recent advances in markerless hand tracking allow Head-Mounted Devices (HMDs) to capture 3D hand motion without external hardware, improving comfort and enabling mobile VR input.

**Figure 1:**
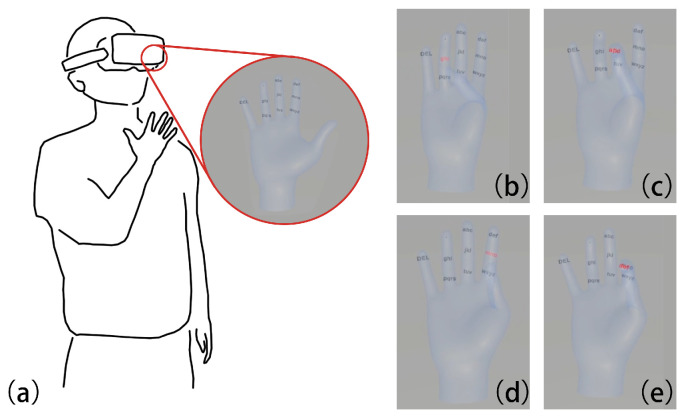
Core concept of FingerType. (**a**) Schematic of the one-handed interaction posture; (**b**–**e**) Sequence of typing the word “hand” by contacting different finger regions. The red highlights indicate the selected character groups and contact positions.

This paper investigates bare-hand, one-handed typing using a VR headset, focusing on four fingers (Index, Middle, Ring, and Pinky) and three phalangeal regions (distal, middle, and proximal), resulting in twelve input zones. We analyze how action types (tap and long press) and finger regions influence thumb-to-finger contact distances, and we assess the operational comfort of each region to guide the design of the FingerType key layout. The proposed interaction concept and a typing example are illustrated in Figure 1.

We then implemented the complete FingerType system with two core components. First, a Temporal Convolutional Network (TCN) processes 3D hand-tracking data to recognize tap events and infer likely target zones; it achieves 94.97% accuracy for tap recognition. Second, an n-gram language model decodes the recognized sequence into words by combining model scores with a beam search over candidate words. The system runs at 72 fps, providing real-time feedback to users actions.

We evaluated FingerType in a VR phrase-transcription study with 24 participants. After six training blocks, FingerType achieved a mean input speed of 10.63 words per minute (WPM). In addition to entry rate, participants required less visual attention and reported lower fatigue with FingerType than with touch input. Performance continued to improve across training blocks, indicating potential for further gains with extended practice.

In summary, we make the following contributions:

- We analyzed thumb-to-finger interaction across 12 zones, focusing on contact distances and comfort ratings. We then used these ergonomic data to optimize the key layout for one-handed input.
- We prioritized accessibility and interaction design by developing FingerType on off-the-shelf consumer VR hardware. Unlike methods requiring custom sensors, we demonstrate that high-precision, eyes-free input is achievable on standard devices without external wearables.
- We proposed FingerType, a bare-hand, one-handed T9 text-entry system for head-mounted devices that uses a TCN to recognize tap events (94.97% accuracy) and an n-gram language model to decode input into words.
- We evaluated FingerType against controller and touch baselines in error patterns, attention demand, subjective perception and input speed, and analyzed its potential for further improvement.

## 2. Related Work

To inform the design of FingerType, we systematically reviewed prior work on text entry in HMDs, hand/finger-gesture text entry, and thumb-to-finger input in VR and similar environments. Table 1 compares FingerType with representative methods. To provide a clearer technical context, we categorize these approaches by sensor type and reported recognition accuracy. This comparison highlights that while many gesture-based methods rely on statistical decoding (marked as ‘-’), FingerType achieves high discrete accuracy (94.97%) using standard VR cameras, matching the reliability of specialized hardware like fingerprint sensors.

### 2.1. Text Entry in HMDs

Text entry is a crucial interaction method for HMDs. Numerous studies have explored various input techniques, including those using physical devices (e.g., keyboards [28], controllers [20], touchscreens [29]) as well as gesture-based [21] and other hand input methods. In VR headsets, text entry often uses virtual keyboards (aim-and-shoot) or index-finger pinch gestures. Virtual keyboards can occlude the view and reduce accuracy, so multi-letter mappings (e.g., T9- or QWERTY-based schemes) have been explored but trade off familiarity and precision [14,16,17,18]. Alternative layouts (e.g., pie-shaped) also address space limits [19,20], yet each approach involves compromises in comfort or accuracy. Pinch-based input suffers from hand jitter (Heisenberg effect), which degrades accuracy and speed, and frequent interactions in VR can cause physical fatigue. Studies also indicate that reducing musculoskeletal strain improves input performance [7,30]. Therefore, our design focuses on minimizing occlusion and alleviating user fatigue, which are core objectives of this work.

### 2.2. Text Entry Through Hand/Finger Gestures

Computer vision and gesture recognition have enabled diverse interaction methods beyond traditional typing. For instance, recent research has successfully utilized optical sensors to detect Greek Sign Language alphabets using deep learning models [31], demonstrating the potential of vision-based systems in interpreting complex hand configurations. Similarly, biosignal-based interfaces, such as Surface-Electromyography (sEMG) readout systems, have been employed to decode muscle activity for draft control applications [32]. While these approaches illustrate the versatility of vision and biosignals for macro-gestures, efficient VR text entry often requires capturing more subtle, rapid finger movements to reduce fatigue. Gesture-based text entry dates back to the late 1970s with data gloves tracking finger motions [33]. Subsequent wearable sensors such as the textile-based Plex and finger-worn Textile Input with Multimodal sensing (TIMMi) enabled eye-free, mobile interaction [34,35], though comfort and scalability remain challenges. Following these developments, modern inertial measurement units (IMUs) are widely used for precise gesture tracking and converting gestures into text input. Single-IMU solutions like LightRing and QwertyRing capture contact and movement for 2D input with an index-finger ring [11,36]. Dual-IMU systems (e.g., DRG-Keyboard) add a thumb sensor to improve relative positioning and support swipe-based virtual keyboard entry on the fingertip [21]. Other body-part input has used wearables such as arm-worn sensors (e.g., TouchEditor), palm/forearm projections (OnArmQWERTY), foot-based methods, or fingernail sensors [6,22,23,24]. Moreover, all these methods require wearable devices, which can lead to issues such as user discomfort and limited battery life, even with wireless Bluetooth connectivity. To address these challenges, we explore text entry using the HMD’s built-in (camera-based) hand tracking in VR, avoiding additional wearables.

### 2.3. Thumb-to-Finger Input

Thumb-to-finger interactions are both natural and precise, offering strong control through tactile and proprioceptive feedback. Previous studies [2,8,10,14] have demonstrated that each finger, or even specific regions of a finger, can function as individual keys. Existing thumb-to-finger systems leverage various sensing modalities—computer vision (e.g., ThumbAir, TipTopTyping), glove or pressure sensors (e.g., DigiTouch, HiFinger), and biometric touch (PrinType)—achieving speeds from 5 to 34 WPM [1,12,13,25,26]. Eyes-free thumb-tip methods like TipText and BiTipText also show promise [27]. However, few studies have systematically characterized fine-grained thumb-to-finger targets across multiple fingers and segments. We study thumb-to-finger input across 12 zones (four fingers × three segments) and evaluate performance and user experience.

## 3. Thumb-to-Finger Typing Behavior Analysis

Current gesture tracking technologies, such as MEgATrack [37], use multi-camera headsets for high-precision, low-latency tracking. However, with typical errors of 11.0 mm (stereo) and 15.7 mm (monocular), accurately distinguishing nearby thumb-to-finger contact regions based on absolute position alone remains challenging. Therefore, we infer user intent from continuous 3D finger motion by analyzing tapping and long-press trajectories across multiple contact positions. We collected 3D hand-tracking data and comfort ratings to guide the design of the FingerType system.

### 3.1. Data Collection for Motion Analysis

#### 3.1.1. Data Collection Setup

**Figure 2:**
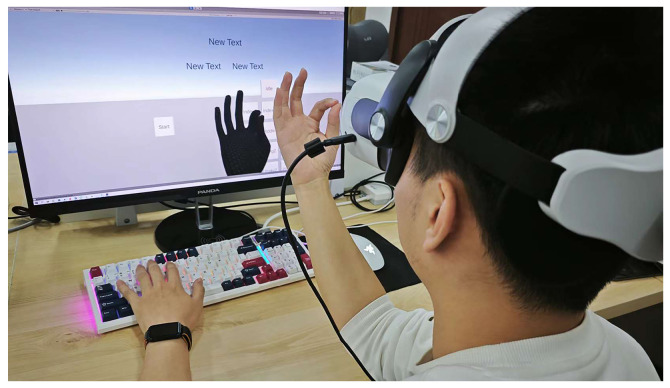
Experimental setup. Participants wore a Meta Quest 2 headset and performed thumb-to-finger taps.

We recruited 13 university students (9 males, 4 females) aged 19–24 (mean = 22.77) with no prior VR experience. Before the experiment, they completed a short training session to familiarize themselves with the equipment and tasks. We used Unity 2022 (Unity Technologies, San Francisco, CA, USA) and the Meta Quest 2 (Meta, Menlo Park, CA, USA) built-in hand tracking to record 3D joint positions. We processed the data offline on a laptop. Table 2 lists the detailed setup. Participants sat in an adjustable chair and held their right hand palm-up throughout the task (Figure 2).

#### 3.1.2. Data Collection Procedure

We relied solely on the Meta Quest 2’s built-in hand tracking, without external optical tracking. Participants performed trials in the 12 predefined regions (defined above) using two actions: a standard tap (brief press) and a long press (press-and-hold for 1 s). The headset continuously recorded 3D hand joint trajectories throughout the session, and participants performed the tasks at their own pace. After the experiment, they rated the comfort of each region on a 5-point Likert scale (1 = very comfortable, 5 = very uncomfortable; higher scores indicate lower comfort).

### 3.2. Analysis of Typing Behavior

#### 3.2.1. Phases of Thumb Movement During Key Press Actions

**Figure 3:**
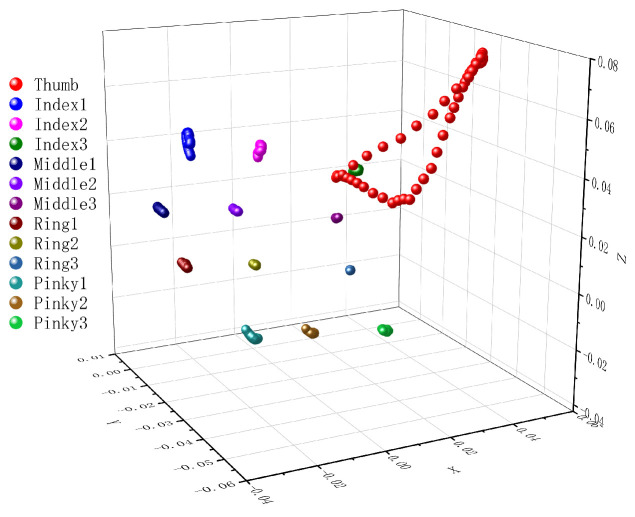
3D trajectory of thumb movement toward Index2.

We collected 325 action records from 13 participants (13 × [2 actions × 12 segments + 1 idle state]). Figure 3 shows the thumb trajectory during a tap on the second joint of the index finger (Index2). The motion can be divided into three phases.

The three phases are ThumbDown (press), Contact, and ThumbUp (release). The Contact phase, when the thumb is closest to the target, is the most informative for distinguishing target zones and is used for input detection. We define the contact distance as the minimum Euclidean distance between the tracked thumb tip and the target finger zone during the Contact phase, and use it as a per-trial summary metric in the following analyses. We verified that all 325 collected records contained these three phases to ensure data consistency for subsequent analysis.

#### 3.2.2. Contact Distance Across Two Tapping Modes

**Figure 4:**
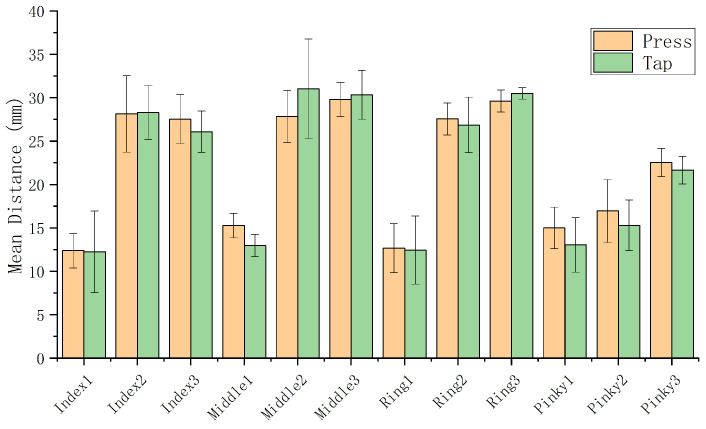
Comparison of contact distances between Tap and Press actions (error bars indicate standard error).

Using the contact distance defined above, Figure 4 shows contact distance differences across finger regions and action states. Repeated Measures Analysis of Variance (RM-ANOVA) revealed a significant difference between standard taps and long presses at Middle1 ($F_{1,12}=16.81,\;p<0.001$), possibly due to joint flexibility or user habits. However, no significant differences were found at other positions. Therefore, except for Middle1, contact distance patterns are largely consistent across tap and long-press actions. Accordingly, we treat tap and long-press inputs identically and extract features from the Contact phase using a unified criterion.

#### 3.2.3. Contact Distance Across Finger and Position Groups

**Figure 5:**
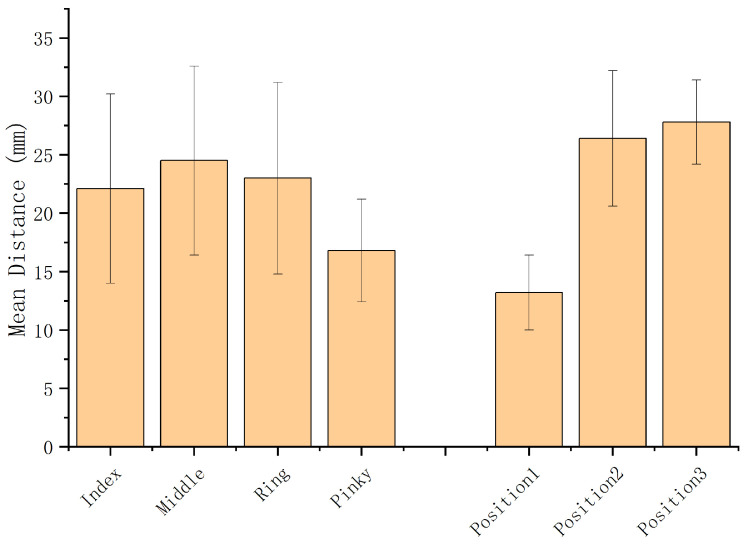
Contact distances across Finger and Position groups (error bars indicate standard error).

Figure 5 shows significant contact distance differences between finger groups (three positions per finger) and position groups (same position across four fingers), based on RM-ANOVA results.

*Finger Group.* Significant differences were found among fingers (Index, Middle, Ring, Pinky) ($F_{3,36}=11.29,\;p<0.001$). The Pinky group had notably shorter contact distances than Index ($F_{1,12}=18.95$), Middle ($F_{1,12}=39.02$), and Ring ($F_{1,12}=24.80$), all p<0.001. Participants noted that lower dexterity in the little finger made thumb interactions at Pinky positions more difficult, increasing task complexity. Accordingly, future systems should avoid mapping complex actions to Pinky positions to improve overall usability.

*Position Group.* Significant differences were also found across positions (Position1–3) ($F_{2,24}=354.73,\;p<0.001$). Position1 had much shorter distances than Position2 ($F_{1,12}=409.96$) and Position3 ($F_{1,12}=924.12$), both p<0.001. Participants feedback indicated that the natural finger flexion when tapping at Position1 made the action easier to perform and more comfortable. Therefore, the shorter contact distance at Position1 is likely related to its higher perceived comfort.

#### 3.2.4. Spatial Distribution of Contact Positions

**Figure 6:**
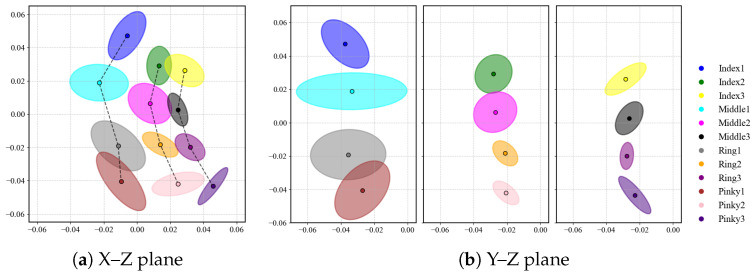
Contact positions projected onto the X–Z (**a**) and Y–Z (**b**) planes.

Figure 6a visualizes contact positions projected onto the X–Z plane. For each finger region, we summarize the contact cloud with a 1σ ellipse (computed from the empirical covariance), which captures the dispersion and principal direction of variability. Across regions, the ellipses align with the expected contact areas on the finger surface, indicating stable tapping locations. Figure 6b compares Position1, Position2, and Position3 in the Y–Z projection. Position1 shows a visibly larger spread than the other two positions, whereas Position2 and Position3 form tighter clusters, indicating more consistent contact locations. This difference may be related to greater posture changes (e.g., finger flexion) when tapping at Position1.

### 3.3. Comfort Evaluation and Design Implications

#### 3.3.1. Comfort Ratings of Tapping Positions

**Figure 7:**
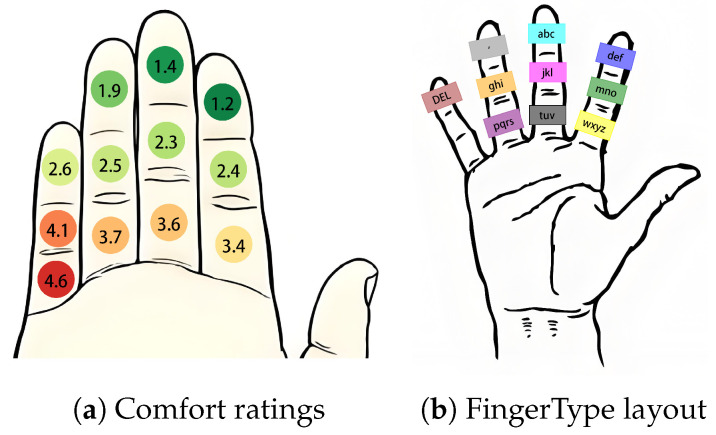
Comfort ratings and system layout. (**a**) Comfort ratings by finger region (1 = very comfortable, 5 = very uncomfortable); (**b**) T9-based key mapping showing letter groups on index, middle, and ring fingers, with delete on the little finger tip.

Figure 7a reports the mean comfort ratings for each finger region (5 = very uncomfortable). Ratings tended to increase (i.e., become more uncomfortable) for regions farther from the thumb and for more proximal targets (Position2 and Position3) compared with the fingertip target (Position1). In post-study comments, participants frequently described Position1 as closer to their habitual thumb-to-finger motion, whereas Position2/3 required maintaining a more flexed posture or reaching closer to the palm. These factors may increase perceived effort during repeated tapping and, thus, reduce comfort.

#### 3.3.2. Design Implications for FingerType

We prioritized a 9-key layout over QWERTY to ensure accuracy and comfort. The limited surface area of fingers cannot accommodate 26 distinct keys without creating tiny, error-prone targets (the “fat finger” problem). By reducing the key count, we restrict input to the most comfortable regions identified in our study, avoiding awkward reaches to the finger roots.

To minimize the learning curve, we adopted the standard ITU E.161 (T9) [38] logical grouping. In determining the physical mapping to finger segments, we assessed two primary strategies found in the literature. Performance-driven approaches (e.g., FingerText [24]) optimize key placement for speed but often break the standard alphabetical order, imposing a high learning cost. Conversely, familiarity-driven approaches (e.g., FingerT9 [14]) prioritize the ITU E.161 layout for memorization but may not explicitly account for the biomechanical strain of specific finger regions.

Therefore, we adopted a balanced strategy: we retained the familiar ITU E.161 logical structure to ensure learnability but optimized the physical placement based on our ergonomic data. We mapped the standard T9 columns to the Index, Middle, and Ring fingers—verified as the most comfortable zones—while assigning the ‘Delete’ key to the accessible Pinky tip (Pinky1). Conversely, we excluded the uncomfortable Pinky base regions (Pinky2/3) due to dual constraints: first, reaching these segments causes excessive strain; second, these extreme poses often cause self-occlusion, leading to unstable tracking. During typing, five candidates are predicted and displayed in real time, with the first preselected by default. A fist gesture toggles the candidate-selection interface (activate/exit). In selection mode, the user selects a candidate by tapping the corresponding finger region (one region per candidate). After confirmation, a space is inserted automatically; tapping the delete region deletes the last character when needed, balancing comfort and efficiency.

## 4. FingerType

FingerType is a mid-air text-entry technique that converts 3D hand keypoint sequences into discrete finger-segment taps and decodes them into words using a T9-style mapping. It combines a motion model for tap detection and key inference with a 5-g language model for candidate generation and ranking.

### 4.1. Tap Detection (Motion Model)

We adopt a TCN as the Motion Model. We chose TCN over traditional Recurrent Neural Networks (RNNs) or Long Short-Term Memory (LSTM) networks for three reasons. First, TCNs compute in parallel, ensuring the low latency needed for real-time VR interaction. In contrast, RNNs process data sequentially, which can introduce delays. Second, TCNs avoid the vanishing gradient problem often seen in recurrent models, making training more stable. Third, dilated convolutions allow us to adjust the receptive field to effectively capture the short, rapid motion of a finger tap. The model processes 20-frame sequences of 3D hand motion data (≈0.28 s at 72 Hz). The optimized TCN, based on Bai et al. [39], consists of four residual blocks with 25 hidden units, kernel size two, and dilation factor two. It outputs a per-frame probability distribution over *K* finger-segment keys and a no-tap (idle) class (K=12). During a tap, the target key probability rises; otherwise, the blank state dominates.

Input features are structured as time series ${\left\{u_{i}\right\}}_{i=1}^{T}$ with T=20, where each $u_{t}={\left\{v_{t,j}\right\}}_{j=1}^{13}$ represents the 3D coordinates of 13 hand keypoints. To reduce viewpoint bias, we project all keypoint coordinates into a local hand-centered coordinate system. We collected a dataset by recruiting 13 participants specifically for data acquisition, each performing 13 gesture classes with three repetitions per class. To increase the number of training samples, we apply a sliding-window scheme that extracts 15 windows per trial, yielding 7605 labeled windows in total for training. The model outputs probabilities over *K* keys plus an additional blank class at each time step.

To evaluate performance of motion model, we conducted an experiment with the same 13 participants described above (10 males, 3 females; mean age: 22.61, range: 20–25), all without prior VR experience. Each participant completed 75 trials (2 tap styles [short/long] × 12 segments + 1 idle, × 3 repetitions), totaling 975 samples. During the experiment, the system output frame-wise class probabilities, which were converted into trial-level decisions using majority-vote temporal smoothing within a fixed time window. Performance was then computed by comparing the smoothed predictions with the ground-truth trial labels. As shown in Table 3, the model achieved 94.97% accuracy, 99.01% precision, and 95.73% recall, demonstrating reliable tap detection.

### 4.2. Text Decoding (Language Model)

We use an n-gram language model implemented with KenLM [40], which estimates the probability of word sequences using conditional probabilities:

(1) $$P\left(w_{1},w_{2},\ldots ,w_{T}\right)=\prod_{i=1}^{T}P\left(w_{i}|w_{i-(N-1)},\ldots ,w_{i-1}\right)$$

Here, $P(w_{i}|w_{i-(N-1)},\ldots ,w_{i-1})$ is the probability of the *i*-th word given the previous N−1 words. To address the issue of zero probability for low-frequency or unseen words due to sparse data, we apply the Witten-Bell smoothing technique [41]. The updated n-gram formula is:

(2) $$P^{*}\left(w_{i}|h\right)=\frac{c(h,w_{i})}{c(h)+T(h)}+\frac{T(h)}{c(h)+T(h)}\cdot P^{*}\left(w_{i}|h^{\prime }\right)$$

where *h* is the history of N−1 words, $c(h,w_{i})$ is the count of the history–word pair, T(h) is the number of distinct words following *h*, and $h^{\prime }$ denotes the backoff history (a suffix of *h*).

We trained a 5-g language model using a 100k English words [42] and 25,000 movie reviews from the Large Movie Review dataset [43]. For efficient decoding, we used beam search with beam width 10 to rank candidate outputs. The resulting language model was evaluated on MacKenzie’s phrase set [44], achieving Top-1 accuracy of 82.67%, Top-3 accuracy of 98.00%, and Top-5 accuracy of 99.78%.

## 5. Materials and Methods

To assess the feasibility of FingerType for VR text entry, we compared FingerType with two widely used VR input methods: controller and touch input [45]. Participants performed a phrase transcription task, and we recorded error behavior, visual attention (gaze/Field of View [FOV]), subjective workload (NASA-TLX), and text entry speed.

### 5.1. Experiment Design

#### 5.1.1. Participants and Apparatus

We recruited 24 college students (17 males, 7 females; mean age: 22.50 years, range: 20–25). All participants were regular QWERTY users and reported no T9 experience in the past three years; therefore, they were considered novices to the tested technique. Participants completed the study seated in an adjustable chair while wearing a Meta Quest 2 head-mounted display. Depending on the condition, input was performed via a single controller (controller condition) or hand tracking (touch and FingerType conditions). All input events and timestamps were logged in real time using the same setup described in Section 3.1.1.

#### 5.1.2. Experiment Design and Procedure

**Figure 8:**
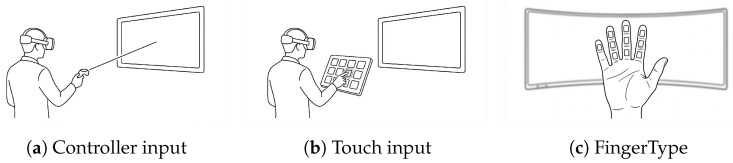
Schematic illustration of the three VR text entry methods used in the experiment. (**a**) Controller input; (**b**) Touch input; (**c**) FingerType.

All three methods used the same phrase display layout (see Figure 8) for consistency. Their main differences lie in interaction distance, input style, and feedback:

- *Controller*: A display panel (3 m × 0.5 m) is placed 3 m in front of the user to present the target phrase. An input panel with large buttons (60 × 20 cm) is operated via ray-casting: users aim a ray at the desired button and pull the trigger to select. Visual feedback is provided by a color change.
- *Touch*: The display panel is the same as in the Controller condition. The input panel is positioned 25 cm in front of user’s chest, and consists of a 4 × 3 grid of medium-sized buttons (8 × 8 × 1 cm). Users touch buttons using a virtual index finger; successful input is indicated by a color change.
- *FingerType*: The display panel is unchanged. The input panel is attached to the virtual hand, with small buttons (2 × 0.5 cm) mapped onto finger segments. Users tap finger segments with thumb to input text, with color change used to confirm each tap.

The study consisted of six blocks. To mitigate fatigue, consecutive blocks were separated by a minimum interval of 12 h and up to two days. In each block, participants transcribed 10 phrases randomly sampled from MacKenzie’s phrase set [44]. Method order was counterbalanced using a 3 × 3 balanced Latin square; the three method orders were repeated across the six blocks (two cycles), and starting orders were evenly assigned across participants. Participants transcribed phrases in blocks, with a 5-min rest after every set of 10 phrases to mitigate fatigue. Prior to Block 1, they completed a brief training session covering all three methods. Each block took about 30–45 min depending on individual entry speed and the scheduled breaks. After completing a block, participants filled out the NASA Task Load Index (NASA-TLX) questionnaire [46] and provided open-ended comments. Overall, we obtained 4320 phrase transcriptions (24 participants × 6 sets per method × 3 methods × 10 phrases per set).

### 5.2. Evaluation Metrics

#### 5.2.1. Words per Minute (WPM)

We computed text entry speed in words per minute (WPM) following MacKenzie [47]:

(3) $$\mathrm{WPM}=\frac{|T|-1}{S}\times 60\times \frac{1}{5}$$

where |T| is the number of characters in the final transcribed string (including spaces), and *S* is the number of seconds from the first input event to the last. The division by 5 converts characters to words under the standard assumption of five characters per word [47].

#### 5.2.2. Normalized Error Ratio ($N_{i}$)

We categorized errors into two log-based types: *repeat errors*, defined as consecutive unintended activations of the same key (e.g., “h-a-a-DEL-n-d”), and *mistake errors*, defined as activating a different (incorrect) key for the intended character (e.g., “h-a-d-DEL-n-d”).

To reduce the influence of letter-frequency distribution on error analysis, we normalized error occurrences. Specifically, for each letter group, we computed a normalized error ratio as follows:

(4) $$N_{i}=\frac{1}{Z}\cdot \frac{X_{i}}{{\mathrm{frequency}}_{i}},\;Z=\sum \frac{X_{i}}{{\mathrm{frequency}}_{i}}$$

where $X_{i}$ is the number of errors for one letter group *i*, and ${\mathrm{frequency}}_{i}$ is the frequency of that group in phrase set.

#### 5.2.3. Attention Coverage Within FOV ($R_{{\mathrm{FOV}}_{\theta }}$)

Eyes-free input is important in text input, as it reduces visual load during multitasking [48]. To evaluate visual attention during transcription tasks, we recorded each participant’s head-gaze direction every second. Note that this is a proxy for gaze and does not capture eye movements independent of head rotation. We then analyzed how attention was distributed between the display panel (target and transcribed text) and the input panel (operation panel) (e.g., controller, virtual keyboard, or virtual hand).

For a given half-angle θ, we define a head-gaze sample as *within FOV* of a panel if the angular deviation between the head-gaze direction and the vector from the user to the panel center is at most θ. The attention coverage is computed as:

(5) $$R_{{\mathrm{FOV}}_{\theta }}=\frac{N_{{\mathrm{inFOV}}_{\theta }}}{N_{\mathrm{total}}}$$

where $R_{{\mathrm{FOV}}_{\theta }}$ is the proportion of head-gaze samples whose directions fall within ±θ of the direction from the user to the panel center, $N_{{\mathrm{inFOV}}_{\theta }}$ is the number of such samples, and $N_{\mathrm{total}}$ is the total number of gaze samples.

## 6. Results

### 6.1. Error Pattern

#### 6.1.1. Error Type Classification

Table 4 reports the proportion of repeat and mistake errors for each method. Overall, mistake errors were more common than repeat errors, suggesting that most errors arose from selecting the wrong key rather than from unregistered presses. FingerType produced the largest share of repeat errors (21.84%). This pattern is plausibly related to FingerType’s delayed visual feedback, which may make it harder to confirm whether a press has been registered. By comparison, the controller and touch conditions provided feedback immediately after each action, and exhibited lower repeat-error proportions (controller: 17.02%; touch: 17.39%).

#### 6.1.2. Spatial Error Distribution

**Figure 9:**
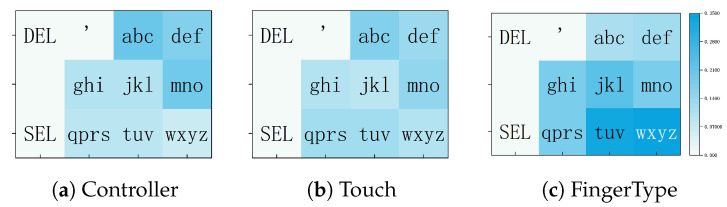
Error distribution for three input methods. (**a**) Controller; (**b**) Touch; (**c**) FingerType. Color intensity denotes error frequency. The key ’ (insufficient data) and leftmost function keys are excluded.

Figure 9 reports the normalized error ratio $N_{i}$ for each letter group (e.g., abc, def). To help interpret the spatial distribution of errors, we refer to Fitts’ law [49], which characterizes pointing difficulty as a function of movement distance and target size:

(6) $$MT=a+b\cdot \log_{2}\left(\frac{D}{W}+1\right)$$

where MT is the mean movement (completion) time, *D* is the distance to the target, and *W* is the target width measured along the movement direction. Although we analyze error rates rather than movement time, increased index of difficulty (larger *D* or smaller *W*) is generally associated with reduced pointing accuracy, leading to higher error rates.

*Controller Input*: All keys are uniformly sized, and the error distribution is consistent with a distance-related pointing difficulty. Due to the relaxed posture of the user’s wrist, the effective ray origin tends to lie below the panel center. The error distribution exhibits a distance-based gradient: central keys (qprs: 9.12%, tuv: 8.71%) closest to the ray origin show the lowest error rates; upper-center keys (ghi: 10.08%, jkl: 10.37%) at moderate distances show slightly higher errors; and edge keys (abc: 20.19%, def: 16.54%, mno: 18.83%) farthest from the origin exhibit the highest error rates.

*Touch Input*: We analyzed errors by key group relative to the (repositionable) panel center, using the panel center as the distance origin. Key groups near the center exhibited lower error rates (ghi: 10.02%, jkl: 9.09%) than groups located farther away (abc: 17.14%, mno: 14.67%, pqrs: 12.43%, tuv: 12.46%). This pattern suggests an association between greater distance from the panel center and higher error rates in the touch condition.

*FingerType*: Error patterns correspond closely with comfort ratings (Figure 7a). For index-finger keys (def: 12.81%, mno: 18.17%, wxyz: 34.18%), errors rise from fingertip to root. A similar trend is seen for middle-finger keys (abc: 10.83%, jkl: 23.15%, tuv: 33.80%). Errors increase near the finger root, which aligns with lower comfort ratings.

#### 6.1.3. Detailed Error Analysis of FingerType

**Figure 10:**
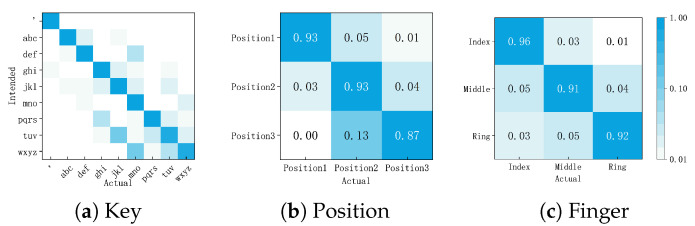
Confusion matrices between intended (rows) and actual (columns) inputs. (**a**) Key confusion; (**b**) Position confusion; (**c**) Finger confusion. Rows are normalized to account for imbalanced intended input frequencies.

Figure 10 shows the confusion matrices between intended and actual FingerType inputs. We summarize the error patterns from two perspectives: (1) finger groups (Index: def, mno, wxyz; Middle: abc, jkl, tuv; Ring: ’, ghi, pqrs) and (2) position groups (Position1: ’, abc, def; Position2: ghi, jkl, mno; Position3: pqrs, tuv, wxyz).

*Fingers are more distinct than positions*: Overall accuracy aggregated by finger group (Index: 96.43%, Middle: 90.56%, Ring: 92.01%) was higher than that aggregated by position group (Position1: 93.40%, Position2: 93.22%, Position3: 86.87%), indicating that participants could more easily distinguish between different fingers than between segments on the same finger.

*Accuracy differed across fingers*: The index finger yielded the highest accuracy (96.43%), while the middle and ring fingers were lower (90.56% and 92.01%). Notably, these two fingers also received lower comfort ratings in Figure 7a. We, therefore, treat reduced comfort as a plausible contributor, although we did not separately model comfort as a predictor of accuracy.

*Lowest accuracy at root segment*: Among segments, Position3 showed the lowest accuracy (86.87%). This segment typically requires greater finger flexion and can introduce more physical strain, which may reduce precision. In addition, FingerType relies on the Oculus hand-tracking pipeline. Because pinch is the primary selection gesture in Oculus interaction, the recognizer may be tuned to be most reliable for pinch-related fingertip configurations. Subtle motions near the finger root may, therefore, provide weaker cues, potentially increasing misclassifications at Position3.

Overall, the observed error patterns align with the comfort ratings (Figure 7a), suggesting that both ergonomics and recognition robustness may jointly affect accuracy.

### 6.2. Attention Demand

**Figure 11:**
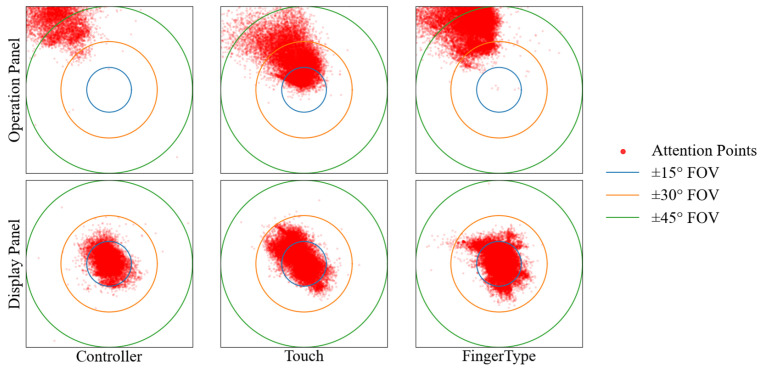
Attention-point distribution for all input methods. Different circle colors indicate the half-angle threshold (blue: ±15°, orange: ±30°, green: ±45°).

We quantify attention allocation as the proportion of samples whose angular deviation θ falls within a given threshold, where θ is computed between the head-gaze direction and the vector from the user to the panel center (Table 5 and Figure 11).

*Controller Input*: Users rarely oriented their gaze toward the operation panel (0.01% within ±15°, 16.54% within ±45°). One likely reason is that the physical controller offers tactile and proprioceptive feedback, so users do not need to visually confirm the input surface. In contrast, gaze remained largely on the display panel (91.59% within ±15°), exceeding both touch and FingerType at the same threshold. Overall, controller input appears to support keeping attention on the text area during entry.

*Touch Input*: Touch produced substantially higher operation-panel coverage (97.47% within ±45°), reflecting the need to visually locate keys and align the hand with the virtual keyboard. At the same time, display-panel coverage within ±15° dropped to 69.47%. Together, these values point to frequent gaze switching between the keyboard and the text region.

*FingerType*: FingerType showed low reliance on the operation panel (0.02% within ±15°), similar to controller input. Display-panel coverage within ±15° reached 84.41%, higher than touch and closer to controller. This may be explained by thumb-to-finger contact acting as a proprioceptive reference for key selection, reducing the need for visual checking of the operation panel.

### 6.3. Subjective Perception

**Figure 12:**
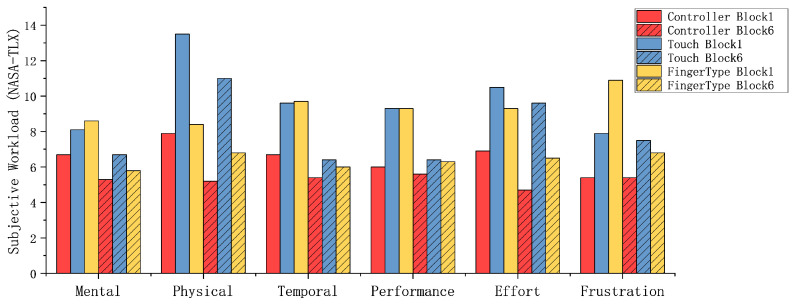
Raw NASA-TLX ratings (0–20) for the six dimensions by input method (shown separately for Block 1 and Block 6). Lower scores indicate lower ratings on the corresponding dimension.

Figure 12 reports raw NASA-TLX ratings on a 0–20 scale for the six dimensions, shown separately for Block 1 and Block 6.

*Controller Input*: Controller was rated lower than the other methods on several dimensions, especially early in practice. In Block 1, we observed significant differences between methods for performance ($F_{2,46}=5.50,\;p<0.01$), effort ($F_{2,46}=6.70,\;p<0.01$), and frustration ($F_{2,46}=15.97,\;p<0.001$). Descriptively, in Block 1 the controller condition received mean ratings of 6.9 on effort and 5.4 on frustration. Participants repeatedly characterized the controller technique as easy to pick up and noted that it “did not require constant attention to hand movements,” which aligns with its lower effort/frustration ratings.

*Touch Input*: Ratings on physical demand differed by input method in both Block 1 and Block 6. A one-way repeated-measures ANOVA showed a significant effect of method in Block 1 ($F_{2,46}=20.61,\;p<0.001,\;\eta_{p}^{2}=0.47$), and the effect remained significant in Block 6 ($F_{2,46}=8.74,\;p<0.01,\;\eta_{p}^{2}=0.28$). As shown in Figure 12, touch was descriptively associated with the highest physical-demand ratings in both blocks. Participants attributed this increased workload to repeated upper-limb movements and the need to visually check hand alignment with the virtual keyboard, which they described as fatiguing over time.

*FingerType*: In Block 1, frustration ratings were descriptively highest for FingerType (Figure 12). Participants commonly attributed this to the unfamiliar key mapping and to difficulty regulating click pressure and dwell time. Within the FingerType condition, Wilcoxon signed-rank tests comparing Block 1 vs. Block 6 showed lower perceived workload for performance (Z=−2.80, p<0.01) and frustration (Z=−2.70, p<0.01). Other dimensions showed smaller changes that did not reach the conventional p<0.05 threshold (mental demand: Z=−2.65; temporal demand: Z=−2.65; effort: Z=−2.04). In comments, several participants noted that “adjusting hand position or wrist angle unexpectedly improved input precision,” and some reported that “the system seemed to learn from prior inputs.” Overall, the Block 1 to Block 6 reductions are consistent with users becoming more familiar with the FingerType technique over practice.

**Figure 13:**
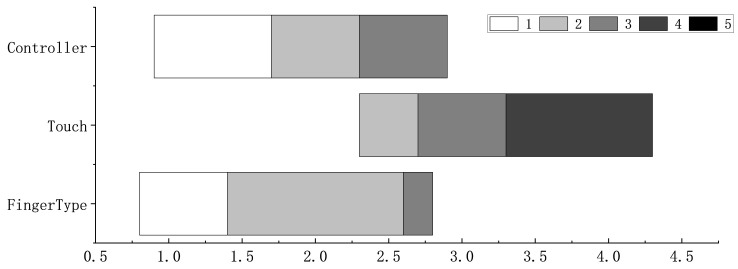
Attention demand for all input methods. The shades correspond to the subjective rating scores indicated in the legend (1 = lowest demand, 5 = highest demand).

Figure 13 shows participants’ self-rated attention demand (5-point scale) for the three input methods. Pairwise comparisons showed that touch required significantly more attention than controller ($F_{1,23}=13.57,\;p<0.01$) and FingerType ($F_{1,23}=20.88$, *p* < 0.001). No difference was found between the controller and FingerType ($F_{1,23}=0.09,\;p>0.1$). These results indicate that touch input imposed higher visual-attentional demands, as participants often needed to look down to confirm hand placement and key alignment on the virtual keyboard, which in turn interrupted immersion. In contrast, controller’s haptic feedback and FingerType’s proprioceptive cues reduced the need for visual checking, allowing participants to keep their attention on the content. Participants commented that eyes-free use was difficult with touch, whereas FingerType felt more intuitive and less visually demanding, supporting a more immersive experience.

### 6.4. Input Speed

**Figure 14:**
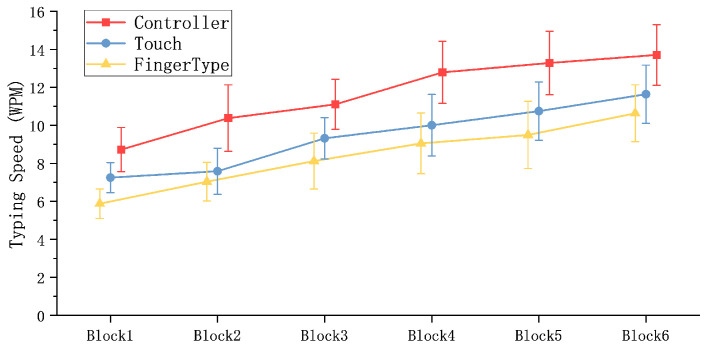
Input speed comparison of three methods (Controller, Touch and FingerType) over 6 blocks.

FingerType exhibited strong short-term learning across the six blocks: WPM increased significantly from 5.88 (SD = 0.77) in Block 1 to 10.63 (SD = 1.49) in Block 6 ($F_{5115}=13.57,\;p<0.001$). This corresponds to an 80.89% increase, which was numerically larger than the gains observed for controller (57.09%) and touch (60.55%). Consistent speed improvements were observed for all three input methods. By Block 6, controller input achieved the highest WPM (13.70, SD = 1.60), followed by touch (11.64, SD = 1.53) and FingerType (10.63, SD = 1.49), as shown in Figure 14. A repeated-measures ANOVA revealed significant differences among methods ($F_{2,46}=35.36,\;p<0.001$), with controller input outperforming both FingerType ($F_{1,23}=37.51,\;p<0.001$) and touch ($F_{1,23}=13.85,\;p<0.01$). In Block 6, the difference between FingerType and touch was not significant ($F_{1,23}=2.00,\;p>0.1$), indicating comparable performance after brief practice.

The model estimates input time at the character level and incorporates the time overhead of recognition errors, which in our case mainly comes from deletion and re-entering characters. We follow the WPM computation in Section 5.2.1: the total input time *S* is approximated as a frequency-weighted sum of per-character times, where the occurrence frequencies are estimated from prior user input logs:

(7) $$\begin{array}{cc} S & =\left|T\right|\cdot \sum_{i=1}^{26}f\left(w_{i}\right)\cdot t\left(w_{i}\right),\\ t(w_{i}) & =P\left(w_{i}\right)\cdot t_{\mathrm{tap}}+\left(1-P\left(w_{i}\right)\right)\cdot \left(t_{\mathrm{tap}}+\alpha \cdot t_{\mathrm{tap}}\right) \end{array}$$

where $P(w_{i})$ is the probability of correctly entering the intended character $w_{i}$ (taken from the diagonal of the row-normalized confusion matrix), $t_{\mathrm{tap}}$ is the average tap time per character, and α is an error-correction factor capturing the additional time incurred when an error occurs.

From Section 5, we use |T|=29.04, $t_{\mathrm{tap}}=807$ ms (=0.807 s), and α=2.28. Following the standard WPM definition in Section 5.2.1, we use |T|−1 in the numerator (here, 29.04−1=28.04), and approximate WPM as:

(8) $$\mathrm{WPM}=\frac{28.04\times 12}{29.04\times 0.807\times \sum_{i=1}^{26}f\left(w_{i}\right)\cdot \left(3.28-2.28\cdot P\left(w_{i}\right)\right)}$$

Using $f(w_{i})$ and $P(w_{i})$ estimated from FingerType trials in Block 6, the model predicts 11.02 WPM, within 5% of the measured value. For a what-if analysis, we increase $P(w_{i})$ at selected locations while keeping the remaining terms unchanged. Improving Index3, Ring3, and Middle3 to 88.53% is enough for the predicted WPM to match touch input; pushing all locations to 96.93% brings the prediction close to the controller condition, indicating that recognition accuracy at these positions is the main bottleneck.

## 7. Discussion

Our VR study shows where FingerType performs well and where it still falls short compared with controller and touch input, and it highlights concrete targets for improvement.

*Input Speed*: FingerType enables efficient, eyes-free, one-handed bare-hand text entry in VR. After six training blocks, FingerType achieved 10.63 WPM, demonstrating fast initial learnability for a controller-free technique. We attribute this rapid improvement to the intuitive nature of the mapping. By adopting the standard T9 layout, FingerType allows users to rely on their existing alphabetical knowledge rather than rote memorization. This significantly reduces the cognitive load during the learning phase, making the gesture-to-word association convenient to acquire. FingerType is comparable to and outperforms several related approaches, such as HiFinger [12] (9.82 WPM after 25 min), FingerT9 [14] (6.09 WPM after five days), and Lee et al. [2] (6.47 WPM after six days). Since these results were obtained under different tasks, protocols, and timing rules, we treat the above comparisons as contextual references rather than direct benchmarks. Future work should examine longer-term practice (e.g., multi-session/longitudinal training) and explore design refinements (e.g., adaptive mappings, personalized layouts, and improved feedback) to further increase entry rate while maintaining FingerType’s eyes-free, one-handed interaction.

*Attention Demand*: Our gaze analysis confirms that FingerType supports eyes-free interaction, as participants focused primarily on the display rather than their hands. We attribute this to proprioception—users naturally know the position of their own fingers without looking. This aligns with findings from DigitSpace [8] and BiTipText [27], which suggest that the hand’s physical features (knuckles and segments) serve as passive haptic cues. These cues allow users to locate targets tactilely. In contrast, virtual touchscreens lack these physical references, forcing users to rely on visual feedback to aim, which explains the higher visual attention demand observed in the touch condition.

*Accuracy and Robustness*: In our study, recognition accuracy is primarily limited by the reliability of the headset hand-tracking signal. Because we implemented FingerType on Meta Quest hand tracking, the absolute error rate and latency we report should be interpreted as device-specific. We also observed failures consistent with camera-based tracking: when fingers are self-occluded or the lighting is unstable, the tracker produces noisier joint estimates, which translates into less stable predictions. Importantly, these errors are not uniformly distributed: a small number of locations (e.g., Index3, Ring3, and Middle3) accounts for a disproportionate share of recognition failures and the resulting speed loss. Future work should improve robustness with more diverse training data, stronger temporal modeling plus lightweight online calibration, and clearer feedback to help novices recover from errors.

*Physical Fatigue*: Sustained mid-air input is known to increase physical load and can lead to “gorilla arm” fatigue [7,30]. This issue also surfaced in our study: during 5–10 min continuous-entry sessions, participants reported noticeable fatigue. While FingerType keeps the hand in a relatively neutral typing configuration, it still requires prolonged arm elevation and repetitive thumb tapping, so discomfort is likely to accumulate over time and with changes in posture. Future work should improve long-session comfort with lower-effort resting postures and explicit rest/resume, optional passive forearm/arm support when available, plus interaction techniques that encourage posture variability through easy repositioning and hand alternation.

## 8. Conclusions

We introduced FingerType, a one-handed VR text-entry method that uses a T9-style layout. By utilizing off-the-shelf commercial hand-tracking, our approach eliminates the need for custom hardware, making the system directly accessible for standard VR setups. This setup allows for efficient, mostly eyes-free typing with less visual attention and physical effort. We also gathered data on users’ tapping habits and comfort, which helps guide future design changes. After a short training period, users reached an average speed of 10.63 WPM. We looked at error patterns to find system bottlenecks and set priorities for improvement. In the future, we plan to adjust key placement, improve recognition and learnability, add ergonomic support to reduce mid-air fatigue, and grow the training dataset to make the system more robust and adaptable. These steps will help make FingerType a practical and user-friendly VR input tool.

## Figures and Tables

**Table 1:** Comparison of FingerType with related text input methods in terms of sensor type, recognition accuracy, and input speed.

Design	Examples	Sensor/Device	Accuracy	WPM
T9 Layout Inputs	FingerT9 [14]	Capacitive Sensor	-	5.42
	Force9 [15]	Pressure Sensor	91.5%	10.8
	PinchText [16]	Conductive Tape	-	12.71
	Lee et al. [2]	Force-sensitive Glove	83.9%	6.47
	**FingerType (Ours)**	**Quest 2 (Camera)**	**94.97%**	**10.63**
QWERTY Layout Optimization	T18 [17]	Touchscreen	-	15.7
	EyeClick [18]	Eye Tracker + Ctrl	-	9.41
	PinchType [10]	Optical Tracking	-	12.54
Redesigning Layout Shape	WrisText [19]	Smartwatch IMU	92.0%	15.2
	HiPad [20]	Controller Touchpad	-	13.57
IMU-based Gesture Tracking	QwertyRing [11]	IMU (Ring)	-	20.59
	DRG-Keyboard [21]	Dual IMU	-	12.9
Input Using Other Body Parts	TouchEditor [6]	Piezoresistive Film	95.4%	6.6
	OnArmQWERTY [22]	Vicon Tracking System	-	20.18
	Wan et al. [23]	Vive Tracker	-	11.12
	FingerText [24]	Capacitive Sensor	97.5%	31.3
Thumb-to-Finger Input Method	ThumbAir [13]	Quest 2 (Camera)	98.2%	13.73
	DigiTouch [1]	Resistive Fabric Glove	-	16.0
	PrinType [25]	Fingerprint Sensor	96.4%	34.22
	TipTopTyping [26]	Camera (MediaPipe)	-	6.15
	HiFinger [12]	Pressure Sensor	-	9.82
FingerTip Micro Gestures	TipText [27]	Vicon Tracking System	-	13.3
	BiTipText [27]	Vicon Tracking System	-	23.4

Note: Bold text indicates the proposed method in this study. ‘-’ indicates methods that rely on statistical decoding or deterministic mapping without a reported discrete recognition accuracy.

**Table 2:** Experimental setup details.

Component	Specification
VR Headset	Meta Quest 2
Tracking System	Built-in Hand Tracking
Development Engine	Unity 2022
Operating System	Windows 10
CPU	AMD Ryzen 7 4800H
GPU	NVIDIA GeForce RTX 2060
RAM	16 GB

**Table 3:** TCN model tap detection results.

	Tap	Not Tap
Detected	896	9
Not Detected	40	30

**Table 4:** Proportion of different error types for all three methods.

Method	Repeat (%)	Mistake (%)
Controller	17.02	82.98
Touch	17.39	82.61
FingerType	21.84	78.16

**Table 5:** Attention coverage $R_{{\mathrm{FOV}}_{\theta }}$ (%) on the operation and display panels within a half-angle θ.

Input Method	Operation Panel			Display Panel		
	±15°	±30°	±45°	±15°	±30°	±45°
Controller	0.01	0.67	16.54	91.59	99.91	99.95
Touch Input	28.40	74.54	97.47	69.47	99.78	99.95
FingerType	0.02	6.55	56.58	84.41	99.82	99.99

## Data Availability

The data presented in this study are available from the corresponding author upon reasonable request.
